# Translation and Initial Validation of the Chinese (Cantonese) Version of Community Integration Measure for Use in Patients with Chronic Stroke

**DOI:** 10.1155/2014/623836

**Published:** 2014-06-04

**Authors:** Tai-Wa Liu, Shamay S. M. Ng, Gabriel Y. F. Ng

**Affiliations:** ^1^School of Science and Technology, The Open University of Hong Kong, Ho Man Tin, Hong Kong; ^2^Department of Rehabilitation Sciences, The Hong Kong Polytechnic University, Hung Hom, Hong Kong

## Abstract

*Objectives*. To (1) translate and culturally adapt the English version Community Integration Measure into Chinese (Cantonese), (2) report the results of initial validation of the Chinese (Cantonese) version of CIM (CIM-C) including the content validity, internal consistency, test-retest reliability, and factor structure of CIM-C for use in stroke survivors in a Chinese community setting, and (3) investigate the level of community integration of stroke survivors living in Hong Kong. *Design*. Cross-sectional study. *Setting*. University-based rehabilitation centre. *Participants*. 62 (*n* = 62) subjects with chronic stroke. *Methods*. The CIM-C was produced after forward-backward translation, expert panel review, and pretesting. 25 (*n* = 25) of the same subjects were reassessed after a 1-week interval. *Results*. The items of the CIM-C demonstrated high internal consistency with a Cronbach's **α** of 0.84. The CIM-C showed good test-retest reliability with an intraclass correlation coefficient (ICC) of 0.84 (95% confidence interval, 0.64–0.93). A 3-factor structure of the CIM-C including “relationship and engagement,” “sense of knowing,” and “independent living,” was consistent with the original theoretical model. Hong Kong stroke survivors revealed a high level of community integration as measured by the CIM-C (mean (SD): 43.48 (5.79)). *Conclusions*. The CIM-C is a valid and reliable measure for clinical use.

## 1. Introduction

Community integration involves promoting interactions with society for those who would otherwise find themselves isolated from it [1–3]. It is the ultimate goal of physical, mental, and drug rehabilitation [4]. After disabling injury or illness it means return to a “normal” lifestyle, and it relates closely to the quality of life of people with chronic illness. For example, a retrospective study by Huebner's group of 45 Americans with traumatic brain injury [5] revealed that increased community participation as measured by the Community Integration Questionnaire (CIQ) [6] significantly correlated (*r* = 0.61, *P* ≤ 0.01) with better quality of life. In another study with Chinese population [7], Kwok and his colleagues interviewed 500 subjects at 3 months after stroke and another 433 subjects at 12 months after stroke and found that community participation as measured by the Chinese version of the London Handicap Scale (LHS) [8] was a stronger predictor of community integration than either functional status or poststroke depression, or quality of life.

In 2001 the WHO published its International Classification of Functioning, Disability and Health (ICF) [9], which classifies functioning in the three domains of (1) body, (2) activities and participation, and (3) contextual factors. The body domain looks at changes in body function and structures; the activities and participation domain looks at the capacity to perform and performance of tasks; and the contextual factors domain looks at environmental and personal factors that influence functioning and disability. Activities and participation are emphasized as the major classification components.

There are several existing measures aiming to capture the multiple facets of community integration and measuring different aspects of the construct. They include the 11-item Reintegration to Normal Living Index (RNLI) [4], the 27-item Craig Handicap Assessment and Reporting Tool (CHART) [10], and the Community Integration Questionnaire (CIQ) [6]. Although these measures all have sound psychometric properties, each is grounded on the developer's concept of community integration. For example, the CHART and CIQ were developed using a concept of “handicap” based on the WHO's International Classification of Impairments, Disabilities, and Handicap published in 1980 [11], while the RNLI was derived from the views of consumers, family members, and health care professionals.

In view of the lack of consistent definition of community integration, McColl et al. [12] had attempted to develop a theoretical model of community integration, the Model of Community Integration, from the view of people with brain injuries. Under the notion of this model, community integration was conceptualized to comprise four factors, including “assimilation,” “support,” “occupation,” and “independent living,” and each had specific indicators consistent with the ICF's concept of “activities and participation.” Based on the Model of Community Integration, McColl et al. developed the original Community Integration Measure (CIM) [13].

The CIM has 10 items with each item soliciting a rating on a five-point scale, giving a minimum score of 10 to a maximum of 50. The CIM has demonstrated good psychometric properties with good internal consistency (Cronbach's alpha = 0.87) [13, 14] and good convergent validity [13] in distinguishing acquired brain injury survivors from healthy adults. The CIM scores also have significant positive correlations with various validated measures including the Community Integration Questionnaire [6], the Satisfaction with Life Scale (SWLS) [15], the Social Provision Scale (SPS) [16], and the perceived mental health quality of life composite score of the SF-12 Health Survey (SF-12) [17]. However, the test-retest reliability of the CIM has not yet been established.

For the factor structure, the original CIM [13] revealed a 1-factor structure measuring a unidimensional construct of community integration. In a replication study, Reistetter et al. [3] reported a 3-factor structure, namely, the “support,” “occupation,” and “independence” factors, and the indicators were clustered differently from the original theoretical model. The discrepancy between the identified factor structures in previous studies warrants for a revisit of the underlying dimensionality that the CIM intended to capture.

Clinicians need a reliable and valid measure consistent with the ICF model to document the most substantive outcome of rehabilitation—community integration—particularly for patients with stroke. Chinese stroke survivors, in particular, are poorly served in this respect. Although the CIM is now widely used with survivors of brain injury [3, 13, 14] and spinal cord injury [18], it has not yet been translated from English into any other languages. Thus, the objectives of this study were (1) to translate and culturally adapt the contents of the English version CIM into Chinese (Cantonese), (2) to report the results of initial validation of the Chinese (Cantonese) version CIM (CIM-C) including the content validity, internal consistency, test-retest reliability, and factor structure in a Chinese setting, and (3) to use the CIM-C to investigate the level of community integration of stroke survivors living in Hong Kong.

## 2. Methods

### 2.1. Translation

The translation and cross-cultural adaptation of the CIM involved the following six steps.

**Step 1**

An expert panel consisting of 5 registered physiotherapists (two with 20 years of clinical experience and three with 3 years of clinical experience in stroke rehabilitation) was set up.

**Step 2**

The English version of the CIM was translated into Chinese by 2 independent bilingual translators whose mother language was Chinese. One of them was a professional translator with no background in medicine or rehabilitation and the other was a physiotherapist with more than 3 years of clinical experience. They independently translated the English version CIM into two initial Chinese drafts (D_1_ and D_2_).

**Step 3**

The two initial drafts (D_1_ and D_2_) and the original English version CIM were reviewed by the same translators involved in Step 2. Any discrepancies identified between D_1_ and D_2_ were resolved by discussion to reach a consensus version (D_1-2_).

**Step 4**

For the backward translation, another 2 independent translators who were blinded to the original CIM were involved. One was a trained translator with no medical or rehabilitation background, and the other was a physiotherapist with more than 3 years of clinical experience. Each translated D_1-2_ into English independently. As no conceptual discrepancies could be identified in the back translation, D_1-2_ was adopted as a Chinese version of the CIM.

**Step 5**

The expert panel then evaluated that Chinese version for equivalence of content, semantics, conceptual, and for any technical discrepancies with the original English version. The version they produced was named the Chinese-CIM-Pilot.

**Step 6**

The Chinese-CIM-Pilot was administered to 5 subjects with stroke to ascertain its fluency, clarity, and comprehensibility. Their feedback was used to make minor amendments, and the final version, CIM-C, was confirmed. (See Appendix I in Supplementary Material available online at http://dx.doi.org/10.1155/2014/623836).

### 2.2. Subjects

62 community-dwelling stroke survivors, with a mean of 7.12 years after stroke, were recruited from among the clients of a local rehabilitation organization in Hong Kong (mean age (SD): 60.71 (6.28) years; 42 men, 20 women) (Table 1). Subjects were recruited if they were over 50 years old, had suffered a single stroke at least 1 year before the start of the study, were able to rise from a chair without any arm support, and had an Abbreviated Mental Test score [19] of 7 or above. Potential subjects were excluded if they had any other comorbid neurological disease (e.g., Parkinson's disease) or an unstable medical condition such as cardiovascular problems that might affect proper assessment.

The subjects were informed about the objectives and procedures of the study and invited to sign a consent form before the experiment. The protocol was approved by the Ethics Committee of the administrative institution and conducted according to the principles of the Declaration of Helsinki for human experiments.

### 2.3. Test-Retest Reliability and Factor Structure

All 62 subjects with chronic stroke were assessed with the CIM-C scale through an individual face-to-face interview. 25 of the subjects (40.3%) were randomly selected by drawing lots for reassessment with CIM-C again after one week to establish test-retest reliability. A 1-week interval was used to minimize chances of subjects' recall of contents from the previous assessment. This period was also considered optimal for avoiding potential occurrences of significant events or changes in their life circumstances that could also impact on their self-perceived balance confidence ratings. Factor structure of CIM-C was assessed by Principal Component Analysis (PCA) on all 62 subjects.

### 2.4. Data Analysis

Quantitative data were analyzed using the Statistical Package for Social Science (SPSS), version 17.0. In the translation process, mode calculation and interjudge percentage agreement were used to measure agreement in the 4 areas of cultural equivalence. Modification was required if any item failed to reach 70% interjudge agreement. In the initial validation process, internal consistency was evaluated using Cronbach's *α* coefficient and item-total correlations. Test-retest reliability was established using the intraclass correlation coefficients (ICCs). For the examination of factor structure, principal component analysis was adopted to extract the factors as the original study by McColl et al. [13] and replication study by Reistetter et al. [3]. Scree plot was used to determine the number of factors retained followed by varimax rotation to optimize the loadings of the extracted factors to enhance the interpretability. For the community integration of subjects was characterized using descriptive statistics.

## 3. Results

### 3.1. Characteristics of the Subjects

There were 42 male and 20 female subjects tested. Their mean age was 60.71 (SD = 6.28) and their mean period since stroke was 7.12 years (SD = 3.12). Their mean BMI was 25.95 (SD = 3.44), and none had a history of falling in the previous 6 months. 39 of them had ischemic stroke, 22 had hemorrhagic stroke, and one with unknown character. The majority of subjects required the use of a walking aid (stick, *n* = 31; small base quadripod, *n* = 5; large base quadripod, *n* = 1), and 42 of them required a companion to walk outdoors (67.74%). 34 claimed to have been involved in exercise routinely for 7 times per week (54.9%).

### 3.2. Internal Consistency

The items of the CIM-C demonstrated high internal consistency with a Cronbach's *α* of 0.84 (Table 2). However, the results suggested that deletion of item 5 would increase the overall Cronbach's *α* of 0.01. The *α* coefficients of the individual items ranged from 0.28 (item 5) to 0.72 (item 9).

### 3.3. Test-Retest Reliability

25 of the subjects participated in the reassessment after a 1-week interval. The CIM-C demonstrated good test-retest reliability as reflected in an ICC of 0.84 (95% confidence interval, 0.64–0.93) (see Table 3). The ICC values for the individual items ranged from 0.34 to 0.88 with item 10 (I have something to do in this community during that main part of my day that is useful and productive) showing the most consistency (ICC = 0.88, 95% CI = 0.73–0.95). Item 2 (I know my way around this community) showed the least repeatability (ICC = 0.34, 95% CI = −0.48–0.71).

### 3.4. Factor Structure

The Principal Component Analysis (PCA) is a method in clustering variables and explaining the variances of a measure [20]. It was used in the original [13] and replication [3] studies of CIM to examine the construct being measured and which items could be grouped. To compare the present findings with the previous studies, the factor structure of the CIM-C was examined by the PCA with varimax rotation. A 3-factor structure of the CIM-C, including “relationship and engagement,” “sense of knowing,” and “independent living,” was revealed in the present study.

The correlation matrix of CIM-C revealed that all 10 items intercorrelated fairly well with correlation coefficients greater than 0.3. The Kaiser-Meyer-Olkin value of 0.79 indicated good sampling adequacy [21], and Bartlett's test was highly significant (*X* ^2^ (45) = 245.85, *P* ≤ 0.001); thus, PCA was appropriate. After the factor extraction, the number of factors retained was determined by scree plot. It was noted that the extraction of 3 and 4 factors provided very similar estimates of the amount of variance in the indicators accounted for by the construct being measured. In other words, the degree of which a standard score increased in community integration associated with standard score increased in the indicators was very similar in the 3-factor model and 4-factor model. Thus, two, three, and four factor solutions were attempted and it was revealed that the 3-factor structure explained the highest percentage of variance and it generated the most meaningful factors (Table 4). The loadings of all 10 items in the 3-factor structure exceeded the recommended value 0.4 [22] and ranged from 0.64 to 0.86, with 67.17% of the total variance explained.

### 3.5. Community Integration of Stroke Survivors in Hong Kong

Table 5 shows the mean score for each item in the CIM-C and its standard deviation. The CIM-C ratings ranged from 28 to 50, and the mean score was 43.48 (SD = 5.79). The item with the highest score (4.58 ± 0.66) was item 3 (I know the rules in this community and I can fit in with them) and the item with the lowest score (3.93 ± 1.14) was item 10 (I have something to do in this community during the main part of my day that is useful and productive).

Table 6 compares the mean CIM-C scores of subjects with different selected characteristics. Independent* t*-tests revealed no significant difference in the overall CIM-C scores of subjects with different gender, right or left side hemiplegia, with or without ankle-foot orthoses, or requiring or not requiring a companion to walk outdoors.

## 4. Discussion

This study has produced the first translation of the CIM into Chinese. It revealed the good internal consistency and excellent test-retest reliability of the Chinese version of CIM. In addition, this study extended the findings of previous investigations on the level of community integration of people with various chronic illnesses, including acquired brain injury [13], traumatic brain injury [14], and spinal cord injury [18], by investigating the level of community integration of Chinese stroke survivors in Hong Kong using a measure coherent with the ICF model. Our final 3-factor structure was comparable to those suggested by Reistetter and colleagues [3] for use in patients with brain injury, and our model was also consistent with the original theoretical model. The people with chronic stroke living in Hong Kong were reported to have high level of community integration (mean (SD): 43.48 (5.79)).

### 4.1. Internal Consistency

Internal consistency means that the items measure the same traits of the construct and the subjects' responses are consistent across all 10 items. The Cronbach's alpha of 0.84 is consistent with those generated in previous studies of people with acquired brain injury (0.87) [13, 14]. The individual CIM-C items demonstrated acceptable item-total correlations (*r* > 0.30) [23], except for item 5 (I can be independent in this community; *r* = 0.28). The greatest increase in Cronbach's alpha would result from deleting item 5, but its removal would increase the alpha by only 0.01. Thus, all individual items were considered worthy of retention.

### 4.2. Test-Retest Reliability

The excellent test-retest reliability of the CIM-C indicates a high degree of repeatability for clinical use. The 1-week interval was considered appropriate to exclude memory effects while minimizing the possibility of significant events occurring in the interim. Having the same examiner administered the test may have contributed to the excellent test-retest reliability.

Although item 2 (I know my way around this community) showed only fair agreement between the two measurements, retention of item 2 did not impair the overall reliability of the instrument. The item's low apparent repeatability may be due to unspecified measurement error, failure to interpret the concepts “know my way around” and/or “community” consistently, or real changes in the self-perceived level of community integration. Despite the test-retest interval of only one week, one cannot completely exclude the possibility of substantial events affecting an individual's level of community integration. Furthermore, the concepts expressed in item 2 may not have been considered as concrete as other items having high ICC values such as item 7 (there are people I feel close to in this community) and item 10 (I have something to do in this community during the main part of my day that is useful and productive).

### 4.3. Factor Structure

In the present study, the PCA revealed a 3-factor structure and the findings were highly consistent with the Model of Community Integration that the original CIM [13] was based on. The high consistency on the Model of Community Integration of CIM across the Western population and Hong Kong Chinese population may suggest that the construct of community integration was comparable between the Western society and Chinese society in Hong Kong. This phenomenon might be due to the multicultural environment in Hong Kong. Based on the cross cultural validation for use in patients with stroke, the present study could further build up the evidence of the factor structure of CIM-C.

In the present study, the first factor identified was “relationship and engagement” which comprised aspects of social relationships and activities. The first factor consisted of items 7–10, in which items 7 and 8 involved aspects of social relationships and 9 and 10 involved aspects of social activities. Item 9 (There are things that I can do in this community for fun in my free time) and 10 (I have something to do in this community during the main part of my day that is useful and productive) were included under the “occupation” domain in 3-factor structure suggested by Reistetter et al. [3]. However, it would be more appropriate to include both items 9 and 10 in the first factor of the present study (i.e., social activity and engagement) due to the cultural differences between these 2 studies. It was because the common concept of people in Hong Kong considered that having interpersonal relationships and meaningful daytime engagement was equivalent to having social support and occupation in the community.

The second and third factors emerged in the present study were “sense of knowing” and “independent living,” respectively, which were different from “occupation” and “independence” domains suggested by Reistetter et al. [3]. The second factor (i.e., sense of knowing) consisted of items 1–3 which involved aspect of “sense of knowing” and belonging to the community; while the “occupation” domain suggested by Reistetter et al. [3] consisted of aspects of productive work and independence. The third factor emerged in this study (i.e., independent living) consisted of items 4–6 which involved aspects of independence, living situation, and sense of being accepted; while the “independence” domain suggested by Reistetter et al. [3] involved aspects of orientation and independence. Although discrepancies of clustered indicators existed between the present study and those of Reistetter et al. [3], all our clustered indicators were consistent with the Model of Community Integration [12]. Such discrepancies of clustered indicators between different studies could be explained by the fact that factors represented by two or three indicators could be highly unstable across replications [24].

### 4.4. Level of Community Integration of Hong Kong Stroke Survivors

Our results showed that the overall level of community integration (mean (SD): 43.48 (5.79)) was comparable to those reported among people with spinal cord injury (SCI) [18] (mean (SD): 42.50 (5.80)) but higher than those of people with traumatic brain injury (TBI) (mean (SD): 39.40 (8.10)) [14] and acquired brain injury (ABI) (mean (SD): 28.80 (7.70)) [13]. The study found that people with stroke, like those with SCI, have a high level of community integration as measured by the CIM. The high CIM scores in our subjects could be explained by several reasons. 55 percent of our subjects claimed to exercise 7 times per week, and this group of patients would be more active to join the community activities. In De Wolf's study [18], 60% of the subjects with SCI had a low impairment levels (tetraplegia ASIA D or paraplegia), and all the subjects in our present study had high level of functional mobility. In addition, the functional mobility of our subjects with stroke, similar to those of patients with SCI, can mostly be compensated for by efficient use of assistive devices, walking aids, and/or a wheelchair. For people with brain injuries, the impaired cognition and emotional and personality changes following traumatic brain injuries may underlie their low average CIM scores. Associated memory loss, problem solving skill deficits, impaired social relationship skills, emotional distresses, and impaired self-esteem could lower their CIM scores in the habits of living and self-evaluation aspects.

### 4.5. Limitations

In the present study, the initial psychometric properties of the CIM-C were established using community-dwellers aged over 50 years but with good functional mobility. However, the generalization of the present study was only limited to those fulfilling our inclusion and exclusion criteria. In addition, the sample size of 62 was barely enough for principal component analysis. Further tests of the applicability of the CIM-C to people with other chronic diseases are therefore recommended to further explore the factor structure of the CIM-C.

## 5. Conclusions

Assessing the level of community integration is essential in evaluating rehabilitation outcomes for people with chronic disease. Cross-cultural adaptation of a validated instrument for quantifying community integration can help to build up an internationally comparable body of scientific knowledge. The findings of the present study showed that the CIM is a culturally relevant and a reliable and valid instrument for assessing the level of community integration of Chinese population with stroke in Hong Kong. The instrument is user friendly and requires only a basic level of literacy (not to be assumed among elderly Chinese populations) to conduct, and it could be completed within 3–5 minutes.

## Supplementary Material

The original 10-items Community Integration Measure (CIM) had been validated and used in patients with chronic illnesses including brain injury and spinal cord injury. The translated 10-items Chinese (Cantonese) version of CIM was validated by the use of patients with chronic stroke in the present study.

## Figures and Tables

**Table 1:** Characteristics of the subjects (*n* = 62).

Characteristics	Value, mean ± SD (range)
Age	60.71 ± 6.28 (50–77)
Gender, number	
Male/female	42/20
BMI (kg/m^2^)	25.95 ± 3.44 (18.76–34.77)
Cause of stroke, number	
Ischemic	39
Hemorrhagic	22
Unknown or mixed	1
Hemiplegic side, number	
Left/right	27/35
Years since stroke	7.12 ± 3.12 (1.95–16.86)
Number of falls in past 6 months, number	
0	62
1	0
≥2	0
Use of AFO, number	2
Mobility status, number	
Unaided	25
Stick	31
SBQ	5
LBQ	1
Need a companion to walk outside, number	42
Habit of regular exercise (times/week), number	
0	2
1–3	16
4–6	10
7	34

BMI: body mass index; AFO: ankle-foot orthosis; SBQ: small base quadripod; LBQ: large base quadripod.

**Table 2:** Internal consistency of the CIM-C.

	Corrected item-total correlation	Alpha if item deleted
(1) I feel like part of this community, like I belong here.	0.68	0.81
(2) I know my way around this community.	0.51	0.83
(3) I know the rules in this community and I can fit in with them.	0.43	0.83
(4) I feel that I am accepted in this community.	0.60	0.82
(5) I can be independent in this community.	0.28	0.85
(6) I like where I am living now.	0.45	0.83
(7) There are people I feel close to in this community.	0.38	0.84
(8) I know a number of people in this community well enough to say hello and have them say hello back.	0.59	0.82
(9) There are things that I can do in this community for fun in my free time.	0.72	0.81
(10) I have something to do in this community during the main part of my day that is useful and productive.	0.72	0.80

Note: Cronbach's *α* coefficient for the entire CIM-C equals 0.84.

**Table 3:** Test-retest reliability of the CIM-C (*n* = 25).

Test-retest reliability					
Item	Mean score		ICC	95% CI	
	1st	2nd		Lower	Upper
(1)	4.48	4.44	0.73	0.39	0.88
(2)	4.36	4.48	0.34	−0.48	0.71
(3)	4.36	4.48	0.51	−0.10	0.78
(4)	3.92	4.24	0.72	0.37	0.88
(5)	4.00	4.12	0.60	0.11	0.82
(6)	4.44	4.36	0.79	0.53	0.91
(7)	4.28	4.08	0.83	0.62	0.92
(8)	4.48	4.40	0.76	0.47	0.90
(9)	4.04	4.20	0.62	0.16	0.83
(10)	3.84	4.00	0.88	0.73	0.95

Total CIM-C score	42.20	42.80	0.84	0.64	0.93

**Table 4:** Rotated factor matrix of the CIM-C based on the principal component analysis with varimax rotation.

Item		Factor		
		1	2	3
(8)	I know a number of people well enough to say hello and have them say hello back	0.84		
(10)	I have something to do in this community during the main part of my day that is useful and productive	0.73		
(7)	There are people I feel close to in this community	0.71		
(9)	There are things that I can do in this community for fun in my free time	0.60		
(3)	I know the rules in this community and I can fit in with them		0.86	
(2)	I know my way around this community		0.74	
(1)	I feel like I am part of this community, like I belong here		0.64	
(4)	I feel that I am accepted in this community			0.78
(6)	I like where I am living now			0.69
(5)	I can be independent in this community			0.64

	Eigenvalues	2.72	2.25	1.76
	Variance explained (%)	27.15	22.46	17.55

**Table 5:** Mean and standard deviation (SD) of CIM-C item scores.

Items	Mean	SD
(1) I feel like part of this community, like I belong here	4.53	0.86
(2) I know my way around this community	4.48	0.74
(3) I know the rules in this community and I can fit in with them	4.58	0.66
(4) I feel that I am accepted in this community	4.03	1.08
(5) I can be independent in this community	4.37	0.96
(6) I like where I am living	4.45	0.96
(7) There are people I feel close to in this community	4.33	0.80
(8) I know a number of people well enough to say hello and have them say hello back	4.48	0.80
(9) There are things that I can do for fun in my free time	4.27	0.94
(10) I have something to do during the main part of my day that is useful and productive	3.93	1.14

Total score	43.48	5.79

**Table 6:** Mean CIM-C scores of subjects with different characteristics.

Total sample (*n* = 62)	Mean (SD)
Gender	
Males (*n* = 42)	43.40 (5.76)
Females (*n* = 20)	43.65 (6.01)
Hemiplegic side	
Right (*n* = 35)	43.34 (5.63)
Left (*n* = 27)	43.66 (6.11)
Use of ankle-foot orthosis	
Yes (*n* = 2)	41.00 (8.48)
No (*n* = 60)	43.56 (5.77)
Companion to walk outdoors	
Yes (*n* = 42)	43.35 (6.43)
No (*n* = 20)	43.75 (4.31)
