# Supplementary material for: Translation and Initial Validation of the Chinese (Cantonese) Version of Community Integration Measure for Use in Patients with Chronic Stroke
Source: Biomed Res Int. 2014 Jun 4;2014:623836. doi: 10.1155/2014/623836 (PMC4065661; doi:10.1155/2014/623836)
Supplement: Supplementary file 1 — The original 10-items Community Integration Measure (CIM) had been validated and used in patients with chronic illnesses including brain injury and spinal cord injury. The translated 10-items Chinese (Cantonese) version of CIM was validated by the use of patients with chronic stroke in the present study. [file 623836.f1.docx]

**Appendix I**

**Community Integration Measure (CIM; McColl et al., 2001)**

*For each of the following statements, please indicate whether you agree or disagree:*

1. I feel like part of this community, like I belong here.

[ ] Always agree

[ ] Sometimes agree

[ ] Neutral

[ ] Sometimes disagree

[ ] Always disagree

2. I know my way around this community.

[ ] Always agree

[ ] Sometimes agree

[ ] Neutral

[ ] Sometimes disagree

[ ] Always disagree

3. I know the rules in this community and I can fit in with them.

[ ] Always agree

[ ] Sometimes agree

[ ] Neutral

[ ] Sometimes disagree

[ ] Always disagree

4. I feel that I am accepted in this community.

[ ] Always agree

[ ] Sometimes agree

[ ] Neutral

[ ] Sometimes disagree

[ ] Always disagree

5. I can be independent in this community.

[ ] Always agree

[ ] Sometimes agree

[ ] Neutral

[ ] Sometimes disagree

[ ] Always disagree

6. I like where I'm living now.

[ ] Always agree

[ ] Sometimes agree

[ ] Neutral

[ ] Sometimes disagree

[ ] Always disagree

7. There are people I feel close to in this community.

[ ] Always agree

[ ] Sometimes agree

[ ] Neutral

[ ] Sometimes disagree

[ ] Always disagree

8. I know a number of people in *this* community well enough to say hello and have them say hello back.

[ ] Always agree

[ ] Sometimes agree

[ ] Neutral

[ ] Sometimes disagree

[ ] Always disagree

9. There are things that I can do in this community for fun in my free time.

[ ] Always agree

[ ] Sometimes agree

[ ] Neutral

[ ] Sometimes disagree

[ ] Always disagree

10. I have something to do in this community during the main part of my day that is useful and productive.

[ ] Always agree

[ ] Sometimes agree

[ ] Neutral

[ ] Sometimes disagree

[ ] Always disagree

**Chinese version of Community Integration Measure**

社區整合量法

在下列的問題裡，請選擇同意或不同意

1. 我覺得我是這個社會的一部分，我屬於這個社會

[ ] 經常同意

[ ] 有時同意

[ ] 中立

[ ] 有時不同意

[ ] 經常不同意

2. 我清楚我在這個社會的方向

[ ] 經常同意

[ ] 有時同意

[ ] 中立

[ ] 有時不同意

[ ] 經常不同意

3. 我知道在這個社會的規則，我可以適應它

[ ] 經常同意

[ ] 有時同意

[ ] 中立

[ ] 有時不同意

[ ] 經常不同意

4. 我覺得我被這個社會所接納的

[ ] 經常同意

[ ] 有時同意

[ ] 中立

[ ] 有時不同意

[ ] 經常不同意

5. 我可以在這個社區獨立

[ ] 經常同意

[ ] 有時同意

[ ] 中立

[ ] 有時不同意

[ ] 經常不同意

6.我喜愛我現在居住的地方

[ ] 經常同意

[ ] 有時同意

[ ] 中立

[ ] 有時不同意

[ ] 經常不同意

7. 在這個社會裡有我相熟的人

[ ] 經常同意

[ ] 有時同意

[ ] 中立

[ ] 有時不同意

[ ] 經常不同意

8. 在這個社會裡我認識一了些朋友會跟我打招呼的

[ ] 經常同意

[ ] 有時同意

[ ] 中立

[ ] 有時不同意

[ ] 經常不同意

9. 在這個社會裡我可以在空餘時間做自己喜歡的事

[ ] 經常同意

[ ] 有時同意

[ ] 中立

[ ] 有時不同意

[ ] 經常不同意

10. 在這個社會裡，我每天都可以做到一些有用和有生產力的事

[ ] 經常同意

[ ] 有時同意

[ ] 中立

[ ] 有時不同意

[ ] 經常不同意
